# Management of white spot lesions induced during orthodontic treatment with multibracket appliance: a national-based survey

**DOI:** 10.1007/s00784-022-04454-5

**Published:** 2022-03-25

**Authors:** Manon Isabelle Weyland, Paul-Georg Jost-Brinkmann, Theodosia Bartzela

**Affiliations:** Dept. of Orthodontics and Dentofacial Orthopedics, Center for Oral Health Sciences, Charité – Universitätsmedizin Berlin, corporate member of Freie Universität Berlin and Humboldt-Universität zu Berlin, Aßmannshauser Str. 4-6, 14197, Berlin, Germany

**Keywords:** White spot lesions, Prevention, Demineralization, Multibracket appliance, Orthodontics, Fluoride

## Abstract

**Objectives:**

The study aimed to survey current strategies against enamel demineralization during multibracket therapy (MBT) and guide a prevention concept based on existing scientific evidence.

**Materials and methods:**

The survey comprised questions on the prevention and management of white spot lesions (WSL). The questionnaire was sent via email to orthodontists working in practices and universities throughout Germany. The analysis involved descriptive statistics using the chi-square test (*p* < 0.05).

**Results:**

A prevention protocol was used before MBT by 80.6% of the participants. Less than a quarter of the participants regularly applied topical fluoride (gel or varnish) during MBT. According to the respondents’ assessment, the prevalence of WSL during MBT is 11.6%, mainly observed in 12- to 15-year-old male patients. Orthodontists graduating after 2000 tended to recommend and apply fluoride-containing materials more often than their senior colleagues (*p* = 0.039). Participants from private practices applied fluoride varnish or gel more frequently than those from university clinics (*p* = 0.013). Fluoridation was the most common (70.7%) treatment for WSL after MBT, followed by resin infiltration (21.2%). The majority (80.9%) of the participants favor a guideline for preventing WSL.

**Conclusions:**

WSL prevention during MBT is challenging. Males in puberty are predominantly affected. Younger orthodontists are more concerned about the prevention of WSL during MBT.

Clinical relevance.

The non-negligible prevalence of individuals with WSL emphasizes the need for dental education and health care reform. This would help to implement standardized procedures and establish innovative applications.

## Introduction

White spot lesions (WSL) are chalky, opaque areas on the tooth surface that develop over months and correspond to the earliest clinical signs of dental caries formation [1]. Caries is defined as a dynamic disease process [2]. Pathological factors such as acid-forming bacteria, salivary dysfunction, and frequent intake of fermentable carbohydrates lead to enamel demineralization. A dynamic reversal process occurs due to the presence of protective factors such as antibacterial agents, sufficient salivary secretion, remineralizing ions, and dietary selected nutrients [1–4]. The balance of these competitive factors can be altered leading to a caries process or arrest. A sugary diet favors acidic pH values and, together with low calcium and inorganic phosphorus concentrations in the dental biofilm, eventually inhibits enamel remineralization [5].

WSL, as an initial sign of this imbalance, have a microscopic structure of two zones: a surface zone (30 µm) and the lesion body. As the surface zone is in direct contact with saliva, remineralization by calcium, inorganic phosphate, and fluoride from saliva can occur more quickly, allowing the minerals to reincorporate into the enamel. However, the lesion body is the most demineralized zone and has a 5 to 25% pore volume. The lesion can progress further in this zone, resulting in additional lesions in the surface zone, allowing the acids to diffuse more quickly into the enamel. If the demineralization process continues, a cavitated enamel surface appears [1, 6].

Beyond the primary focus on oral functional improvement, orthodontic treatment also aims to improve esthetics, which increases the self-confidence and general well-being of a patient. However, as fixed orthodontic appliances facilitate plaque accumulation and complicate tooth cleaning, such treatments pose a risk of provoking WSL and its associated negative esthetic, financial, and health implications [7]. Several studies reported a rapid evolution of WSL in the first weeks of multibracket therapy [8, 9], with an increased prevalence of up to 40% within the first 6 months of treatment [9]. The incidence of new WSL is positively correlated with the duration of multibracket therapy [10, 11]. Consequently, WSL can compromise the orthodontic treatment outcome, forcing premature bracket removal.

Few studies currently provide methods to prevent WSL in orthodontic practices [12–17]. Many practitioners deliver primary preventive dental care at bracket bonding, based mainly on oral hygiene instructions [12, 13, 15, 17]. Extra measures are usually taken only after the appearance of WSL. Fluoride rinses have been predominantly recommended in various investigations but not sufficiently prescribed by dental practitioners [12–15, 17]. Chlorhexidine (CHX) or toothpaste with high fluoride concentrations was seldom applied [13].

Dental caries remains the most prevalent non-contagious disease, with 2.3 billion afflicted people worldwide [18]. Regarding the current sanitary situation provoked by the Covid-19 pandemic, one can only suppose that the number must have increased in the meantime. Therefore, national and global strategies should promote dental caries prevention measures [18].

The present study was designed to provide information about the current methods used to prevent enamel demineralization during multibracket therapy in German orthodontic university departments and practices. It also aims to compare these methods with the available evidence from the scientific literature. The study’s objective is to guide efficient prevention strategies of enamel demineralization during orthodontic treatment with fixed appliances.

## Materials and methods

### Survey

A cross-sectional study was conducted by the orthodontic department of the Charité – Universitätsmedizin Berlin, Germany. A multiple-choice questionnaire was designed which addressed the following six items:

- 1. Methods and materials used to prevent demineralization at the start of, during, and after multibracket therapy
- 2. Fluoride release of bracket bonding material based on the manufacturer’s report
- 3. Patients’ compliance with oral hygiene regimens and appointment keeping
- 4. Participants’ experience with WSL formation
- 5. Need for a guideline to prevent demineralization during multibracket therapy
- 6. Participants’ professional background

The participation was utterly anonymous so that no practice-related data could be retrieved. The survey was approved by the Ethics Committee of the Charité – Universitätsmedizin Berlin (EA2/196/20).

### Procedure

From October 2020 to December 2020, all persons contacted received an email with an information letter and access to the online survey (Survio.com). To ensure a maximum response rate, reminders were sent once a month. Completed questionnaires were entered on an Excel spreadsheet and imported into SPSS (IBM SPSS Statistics for Macintosh, Version 27.0. Armonk, NY: IBM Corp) for statistical data analysis.

### Participants

German orthodontists working in private practices and universities were the target group. The sample size calculation showed that a minimum of 120 orthodontists could generate representative data. As a low response rate was expected [19], 900 orthodontists were selected at random out of the 2,543 members listed in the DGKFO (Deutsche Gesellschaft für Kieferorthopädie e.V./ German Orthodontic Society) index. Of these 900, only 584 were considered valid. Exclusion criteria were members being retired or practicing abroad.

Another 127 orthodontists from university clinics, professors, senior dentists, scientific staff members, and residents with a valid email address, available on the university’s website or by departments’ managers or secretaries, were contacted to participate in the study.

In addition, the associations KFO IG (Professional Association for German Orthodontists), GMSCKFO e.V. (Society Master of Science Orthodontics e.V.), and KFO BB (Society for Orthodontics of Berlin and Brandenburg e.V.) have kindly forwarded the questionnaire to their members.

Due to the distribution mode, it was not possible to accurately determine the recipients’ number. We estimated that around 711 orthodontists were contacted.

### Statistical analysis

It was assumed that more than 63% of the participants would favor a guideline for preventing WSL. This proportion was set for a sample size of 120 participants to prove that more than 50% are in favor of a guideline. With a sample size of 120, the one-sided binomial test has a power of at least 80% to reject the null hypothesis that only 50% or less of the participants would like to have a guideline on the prevention of WSL. The one-sided binominal test calculated the observed frequency at the significance level of α = 0.025 against 50%. In addition, a two-sided 95% confidence interval was calculated. The sample size was calculated with nQuery version 8.6.0.0.

The analysis involved descriptive statistics, frequency distribution (relative and absolute frequencies), and cross-tabulation. All participants were clustered into two groups concerning their graduation year, distinguishing between senior participants who graduated before 2000 and younger participants who graduated during/after 2000. According to the workplace, another regrouping was carried out, i.e., private practice, university, and a combination of both. Statistical comparisons between the groups and the questions about the treatment of demineralization before, during, and after multibracket therapy were done with Pearson’s chi-square test using SPSS software (IBM SPSS Statistics for Macintosh, Version 27.0. Armonk, NY: IBM Corp). The significance level was set at *p* < 0.05 for all statistical analyses.

## Results

### Participants

This study employed an external online survey. Until December 2020, 156 questionnaires had been completed. One participant from Switzerland had to be excluded. Participants with graduation year before 2000 were 39 (25.2%), and the remaining 116 participants (74.8%) graduated during/after 2000. Of the participants, 139 (89.7%) completed their orthodontic training in Germany and 16 (10.3%) abroad. There was a total of 29 of the orthodontists (18.7%) working in a university, nine (5.8%) in a combination of university and private practice, and 117 (75.5%) in private practice. Most participants (73%) practiced in cities (> 100,000 inhabitants), 23% in rural areas, and 4% in both urban and rural areas.

**Fig. 1:**
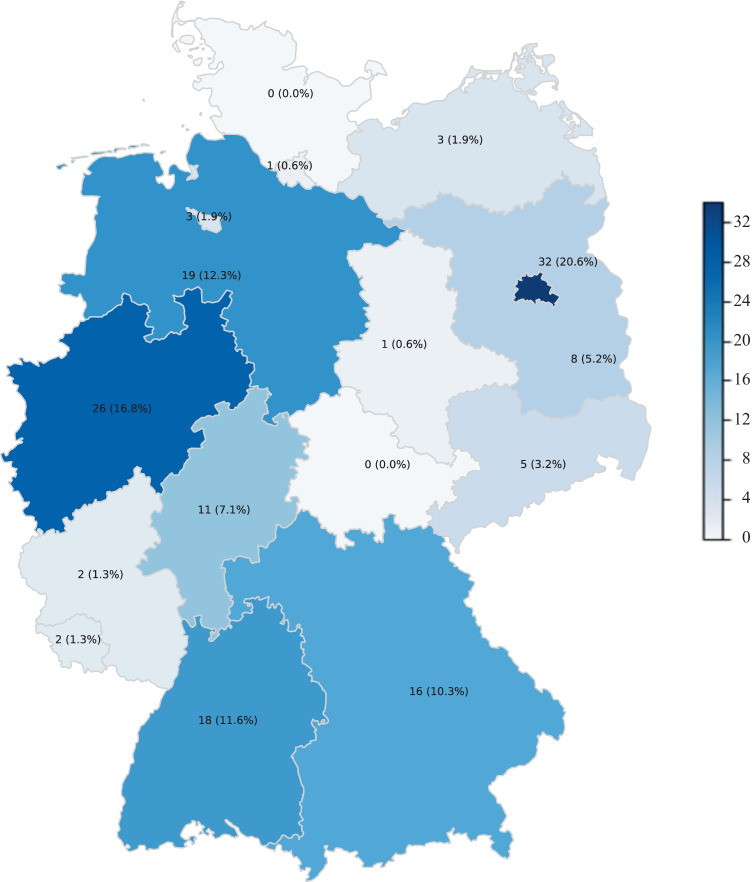
Geographical distribution of the participants (in parenthesis: % of all respondents). The darker shades of blue correspond to the federal states with most participants

Participants from 14 out of 16 all federal states completed the survey, except Thuringia and Schleswig–Holstein (Fig. 1). The highest participation was in Berlin (20.6%), followed by North Rhine-Westphalia (16.8%) and Lower Saxony (12.3%). However, the number of participants did not reflect the number of inhabitants in the respective federal states (Fig. 1).

### Practice protocol

Oral hygiene status registration and a prevention protocol were carried out by 80.6% of the participants at the beginning of multibracket therapy. The prevention protocol and frequencies (% of participants) are presented in Table 1. Most of the participants (73.5%) consistently implement oral hygiene instructions during multibracket therapy. Flossing is more often recommended than an electric toothbrush. Multibracket therapy is started by 68.7% of the participants only when patient compliance with oral hygiene is achieved. In case of oral hygiene deterioration, 21.1% responded that they consistently interrupt the multibracket therapy (Table 1), following the state health insurance recommendations.

**Table 1:** Frequencies (% of participants) for measures and materials used to prevent demineralization at the start of and during multibracket therapy

Protocol for WSL prevention during multibracket therapy	Always (%)	Usually (%)	Sometimes (%)	Never (%)
Education of the current oral hygiene situation	82.9	16.4	0.0	0.7
Oral hygiene instruction	73.5	23.1	2.7	0.7
**Advice of**:				
Electronic toothbrush	17.8	37.0	32.9	12.3
Flossing	83.0	10.9	5.4	0.7
Professional tooth cleaning	54.5	27.6	15.2	2.8
Dietary advice	33.1	20.0	33.1	13.8
Saliva germ count	0.7	1.4	4.9	93.0
**Application of**:				
Fluoride gel	45.1	25.4	17.6	12.0
Fluoride varnish	26.8	28.2	25.4	19.7
Fluoride foam	2.3	0.0	12.0	85.7
CHX varnish or gel	7.2	11.6	39.9	41.3
**Application of:**				
Sealant around brackets, before bracket placement	39.0	14.7	9.6	36.8
Sealant around brackets, after bracket placement	18.6	15.0	20.0	46.4
Lingual appliance or clear aligner instead of conventional labial MB for patients with HCR	11.0	10.3	48.3	30.3
Start MB only when adequate oral hygiene is in place	68.7	25.2	5.4	0.7
**In case of deterioration:**				
Referral to the general dentist	40.7	33.1	19.3	6.9
Warning letter to parents/guardians	48.6	24.7	19.2	7.5
Compromise orthodontic treatment outcome	18.6	37.2	42.1	2.1
Continue treatment with a removable appliance	6.2	22.6	56.8	14.4
Early removal of the MB	21.1	35.4	42.9	0.7

*HCR* high caries risk, *MB* multibracket

Participants from private practices and those working in both university and private practices often use flat surface sealant application before bracket placement (*p* = 0.046). The younger participants included professional tooth cleaning as part of the prevention protocol more often than senior participants (r =  − 0.12, *p* = 0.017). In deteriorating oral hygiene, the younger participants referred patients to the general dentist more often than their senior colleagues (r =  − 0.18, *p* = 0.006).

### Topical materials

If fluoride is applied, it is mainly at the start of the treatment. This applies only to about half of the participants, as shown in Table 2. Later fluoride applications are only carried out by a quarter or less of the participants and not regularly. Only 9% of the participants advised using a 5,000 ppm fluoride toothpaste.

**Table 2:** Frequencies (% of participants) for application and advice of remineralizing agents during multibracket therapy

	Frequency	%
**Application of fluoride gel or varnish:**		
At the beginning of treatment	90	58.1
During professional tooth cleaning	70	45.2
3–4 × a year	39	25.2
2 × a year	30	19.4
Never	12	7.7
At every appointment	9	5.8
Every 6–8 weeks	6	3.9
**Advice of topical materials:**		
Fluoridated toothpaste	134	86.5
Fluoride gel once a week	117	75.5
Fluoride rinse 1–2 × daily	46	29.7
Fluoridated toothpaste with 5000 ppm F	14	9.0
0,06% CHX rinse 1 − 2 × daily	7	4.5
No recommendation	5	3.2
**Preventive measures offered as private supplementary services:**		
Sealant around the brackets	120	77.4
Application of F or CHX gel or varnish more than 2 × a year	39	25.2
No preventive measures offered	29	18.7

*CHX*  chlorhexidine digluconate, *F*  fluoride

Participants working in private practices and those combining university and private practices apply topical fluoride more often than the participants from the universities (*p* = 0.013). The younger participants recommend topical fluoride materials more regularly than the senior participants (r = 0.23, *p* = 0.004).

Slightly more than half of the participants (57.5%) are more attentive to patients with high caries risk. Generally, these patients receive dental check-ups every 2 to 3 months, including fluoride gel or varnish treatment.

**Fig. 2:**
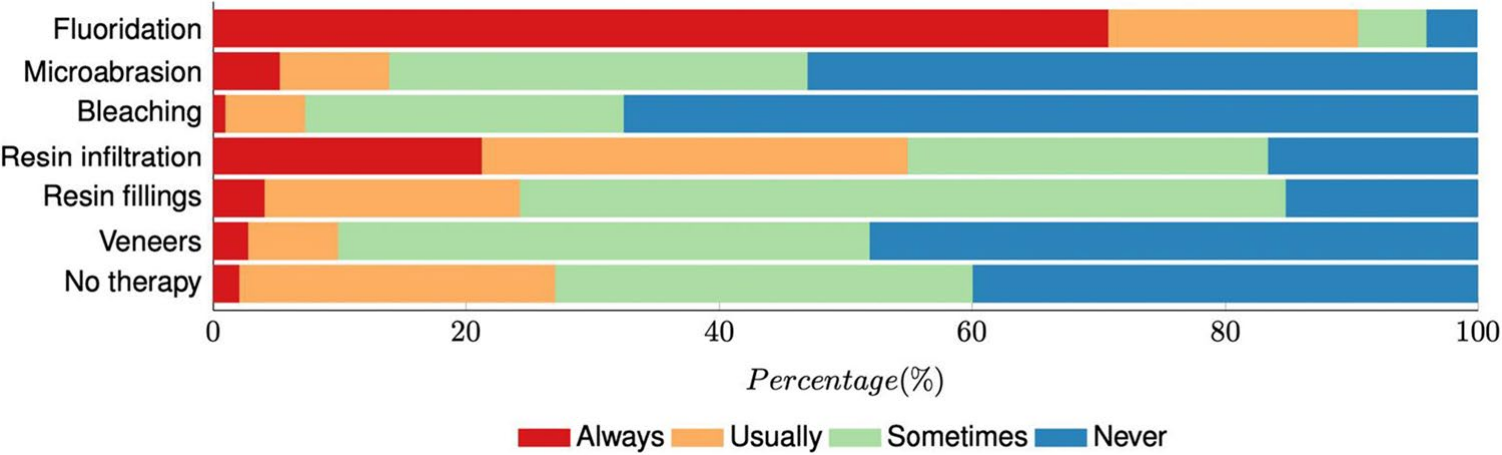
Frequencies (% of participants) of recommended therapies for white spot lesions (WSL) after multibracket therapy

Fluoridation is the therapy of choice for WSL in patients with multibracket appliances after debonding, followed by resin infiltration (Fig. 2). The younger participants recommended more frequent veneers for WSL therapy after multibracket therapy than the senior participants (r =  − 0.27, *p* = 0.033). The participants combining work in university and private practices suggested resin infiltration for WSL therapy after multibracket therapy more often than those working at the university or private practices (*p* = 0.035).

### Bonding materials

Most participants (70.1%) claim to use fluoride-releasing bonding materials (adhesives or cements) for bands and brackets, against 20.8% who prefer not to use fluoride-releasing bonding materials. Fluoride-releasing bonding materials were only occasionally used by 9% of the participants.

### Compliance and motivation

Poor oral hygiene in orthodontic patients is frequently observed and associated with swollen gingiva or plaque accumulation by 54.6% and 43.9% of the orthodontists, respectively. WSL are noticed routinely by 3.4% of the participants and frequently by 11.5%. More than half of the participants (54.7%) stated that the WSL occur more often within the first 9 months of multibracket therapy. Adolescents, especially 12- to 15-year-olds, are more likely to miss dental appointments than adults. Additionally, according to 66.5% of the participants, male patients more often failed to attend their orthodontic appointments than female patients.

Modern and innovative re-motivation methods are seldom adopted. Only a few participants (6.5%) implement reminder methods (text messages) to increase oral hygiene compliance (Table 3). Hardly anyone (0.6%) uses mobile apps for motivation. The younger participants communicate verbal or written information about the current oral hygiene situation more often than their senior colleagues (r =  − 0.16, *p* = 0.043). The university participants and those working at universities and private practices use regular photo documentation to re-motivate patients more often than those working in private practices (*p* = 0.007).

**Table 3:** Frequencies (% of participants) for motivating methods taken during multibracket therapy

Remotivating methods	Frequency	%
Education about the consequences of poor oral hygiene	146	94.2
Information (oral/written) about oral hygiene situation	131	84.5
Show photos of demineralized teeth	115	74.2
Regular photo documentation for re-motivation	80	51.6
Videos/visual demonstration of oral care	56	36.1
Text message reminding to use oral aids (dental floss, mouthwash, etc.)	10	6.5
Chat apps for sharing dental selfies	1	0.6

### WSL

According to most participants (148 out of 155, 7 did not respond), 11.6 ± 11.2% of orthodontic patients develop WSL during multibracket therapy. The median was 6%, and the mode was 5%. Poor oral hygiene is the most detrimental factor for the emergence of WSL (98.7%), followed by poor appointment compliance (83.2%). Less than half of the participants consider the age and flat surface sealant (44% and 41%, respectively) factors influencing the formation of WSL. Only 33% of the participants specified diet as a contributing factor.

The most affected group by WSL during multibracket therapy was mainly male 12- to 15-year-old patients.

### Request for a guideline

Most participants (MD: 80.9%, 95% CI: 73.8% to 86.8%, *p* < 0.001) favor a guideline, and three did not answer this question. Since the confidence interval’s lower limit is higher than 63.3%, the null hypothesis (*p* < 0.05) can be rejected.

Most of the participants (78.3%) expressed an opinion on a guideline. Among them, 91 (58.7%) participants favored a guideline, and 28 (18.1%) refused any form of a structural prevention strategy.

## Discussion

More than half of the children and adolescents in Germany are treated orthodontically. The treatment usually lasts between 2 and 4 years [20]. Orthodontic patients are at an increased risk of developing initial caries during multibracket therapy, leading to a possible serious public health concern [7]. The main objective of this study is to gain insight into preventive measures against WSL during multibracket therapy in Germany and compare these measures with those recommended in the literature to support evidence-based practice.

The estimated WSL prevalence of 11.6% from the survey should not be neglected, although the reported prevalence in the scientific literature is higher (wide range from 23 to 97%) [8–11, 21, 22]. Male adolescents are the most affected individuals, as was also recognized by other researchers [23, 24].

Slightly more than half of the participants give nutritional instructions during multibracket therapy, while 13.8% never employ dietary advice in their prevention protocol (Table 1). The development of WSL can be severely limited by avoiding sugar-sweetened or high-carbohydrate products [23, 25, 26]. Artificial sweeteners, such as stevia, sucralose, and saccharin, even are branded as tooth-friendly, still have a demineralization effect [27]. Nevertheless, the frequency of sugar exposure plays the major role in dental health, not the total sugar intake [25]. Additionally, nutritional instructions can eliminate the risk of material fractures and bracket detachment [28, 29].

An overwhelming majority of the participants run an oral hygiene and prevention protocol at the beginning of multibracket therapy (Table 1). The results strongly resemble those of comparable studies [12, 13, 15, 17]. Consistent with Derks et al. [13], slightly more than half of the participants in this study routinely recommend electric toothbrushes. Powered toothbrushing has been proved better than manual in reducing plaque, gingivitis, pocket depth, and periodontal bleeding in different patient groups, including orthodontic patients [30–32].

Only one-third of participants used visual demonstrations to improve oral hygiene (Table 3). Only a few participants currently use modern technology to motivate their patients consistently. Frequent patient reminders, such as mobile phone applications and text messages, contribute to good oral hygiene during multibracket therapy (Table 4), especially in young adolescents [33–38]. Therefore, as we can see in Table 4, active re-motivation is of great importance.

**Table 4:** Overview of the included systematic reviews and meta-analyses on the prevailing management and treatment strategies for WSL during and after multibracket therapy

References	Type of study	Studies	Participants N / age	Publication yrs	Time of intervention	Prevention material	Follow-up	Effect (95% CI)	Evidence/comment
Benson et al. (2019) [26]	SR	10	1798/any age	2005–2019	During MBT	*Intervention group:* 1. F-containing product (i.e., APF foam, gel, mouth rinse, varnish, HFT) 2. F-releasing glass bead device 3. F-releasing bonding material *Control group:* 1. No treatment with extra F (i.e., placebo foam/gel/mouth rinse/varnish, 1,450 ppm TP) 2. F mouth rinse 3. Light-cured composite resin	17.6 to 24.5 m (until the day of debonding)	*F varnish:* RR = 0.52 (0.14 to 1.93) *12,300 ppm F APF foam:* RR = 0.26 (0.11 to 0.57) *HFT (5,000 ppm F):* RR = 0.68 (0.46 to 1.00)	F varnish (insufficient evidence) 12,300 ppm F (APF foam) prof. applied every 6 to 8 w (low evidence), WSL: APF 13% <—> Placebo 51% 5,000 ppm F (HFT) decreasing the new WSL during MBT (low evidence), new WSL: 5000 ppm 18% <—> 1450–1500 ppm 27%
Tasios et al. (2019) [25]	SR and meta-analysis	23	1473/any age, average age 14.1 yrs	1992–2017	During MBT	*Intervention group:* 1. F-containing product (i.e., varnish, mouth rinse, F-releasing bonding material) 2. Any sealant 3. Active reminder (i.e., mobile-phone, applications and messages) *Control group:* 1. No treatment with extra F (i.e., no varnish, 1,450 ppm TP, F drinking water, resin adhesive) 2. No sealant 3. No reminder	3 to 26.7 m (mid-MBT or directly after debonding)	*F varnish:* RR = − 0.32 (− 0.44 to − 0.21) *Flat surface sealant:* RR = 0.77 (0.63 to 0.95) *Active patient reminder:* RR = 0.44 (0.31 to 0.64)	F varnish: prof. applied every 6 to 12 w reduce WSL incidence: F varnish 27% <—> no varnish 59% (low evidence) Flat surface sealant (insufficient evidence) Active patient reminder: 1x/w to 1x/m reduce WSL incidence: active patient reminder 26% <—> no reminder 60% (low evidence)
Höchli et al. (2017) [27]	SR and meta-analysis	20	942/any age, average age 16.1 yrs	2006–2016	Directly after debonding or up to 14 yrs. in retention	*Intervention group:* 1. CPP–ACP creams (with or without F) 2. External tooth bleaching 3. F-containing product (film, gel, mouth rinse or varnish) 4. RI 5. Miswak chewing sticks 6. Bioactive glass TP *Control group:* 1- 6. No treatment with extra F (i.e., conventional OH, no F TP, 1000–1450 ppm F TP)	4 w to 6.5 m	*F varnish in reducing WSL:* MD = -0.80mm^2^ (-1.10 to -0.50mm^2^) *F varnish in increasing enamel fluorescence:* SMD = − 0.92 (-1.32 to -0.52)	22,600 ppm F varnish or 5% NaF varnish 1x/m promising results in reducing WSL (low evidence), WSL: varnish vs control *p* < 0.0001

*APF* acidulated phosphate fluoride, *CI* confidence interval, *CPP-ACP* casein phosphopeptide amorphous calcium phosphate, *F* fluoride, *HFT* high fluoridated toothpaste, *MBT* multibracket therapy, *MD* mean difference, *m* months, *NaF* sodium fluoride, *nr.* number, *OH* oral hygiene, *Prof. applied* professionally applied, *RI* resin infiltration, *RR* risk ratio, *SMD* standardized mean difference, *SR* systematic review, *TP* toothpaste, *yrs.* years, *w* weeks, *WSL* white spot lesions

Fluoridation was the most frequently mentioned recommendation against WSL therapy after multibracket therapy, followed by resin infiltration (Fig. 2), as also recently reported in a study conducted in the USA [16]. The following most frequently mentioned recommendation was no therapy at all. Refraining from remineralization agents within the first 6 months after debonding may allow the remineralization process to take place on its own [3]. If this is not successful, measures should be implemented depending on the extent and desire for esthetic rehabilitation. Topical material has shown unsatisfactory results to reverse WSL after multibracket therapy [39, 40]. Post-orthodontic WSL differ in localization and structure. Therefore, the remineralization agents can reduce the lesion by potentially inducing enamel staining [41]. Resin infiltration is an alternative not only to arrest the enamel lesions but also as a minimally invasive method to improve the esthetic outcomes after multibracket therapy [42–46]. However, in Germany, resin infiltration is not included in the standard care coverage by the health insurance companies.

Furthermore, self-assembling peptides (SAPs) for preventing demineralization or regenerating the affected enamel are a new prevention approach with promising results [47–51]. SAP P11-4 provides diffusion-based mineralization forming a 3D matrix with the carious lesion [52]. Using SAP P11-4 in combination with fluoride adjacent to the bracket base is more effective than fluoride alone [53]. Furthermore, SAP P11-4 application, before the bracket bonding procedure, did not affect the shear bond strength [54].

The type of fixed orthodontic appliance has a significant impact on oral health. Teeth with lingual appliances are considered less vulnerable to caries than the conventional labial multibracket appliances [55–59]. On the other hand, Lombardo et al. [60] describe increased plaque formation and a higher concentration of *Streptococcus mutans* in the saliva samples of patients treated with lingual appliance. However, salivary flow rate and saliva puffer capacity remain the same. This appears to be beneficial in preventing enamel demineralization, especially lingually where salivary flow or secretion is most abundant. Thus, lingual multibracket appliances are an alternative for patients prone to caries if they are affordable. Likewise, clear aligners seem to promote periodontal health, lower salivary Lactobacilli and Streptococcus mutans levels, and impede plaque accumulation. Hence, oral hygiene might be easier to be maintained [61, 62], and enamel demineralization can be prevented [63, 64]. However, lingual appliances and clear aligners are not the standard care covered by health insurance.

Most of the participants use fluoride-releasing bonding materials, which primarily serve as a reservoir releasing local fluoride. It is important to note that fluoride cannot prevent the formation of biofilms and caries, but only slow down the process [65]. Despite the in between study heterogeneity, the systematic review by Nascimento et al. [66] presented a positive effect of fluoride-releasing bonding materials, with a risk reduction of 58% to WSL formation.

For evidence-based clinical measures for the prevention of WSL during multibracket therapy, recent systematic reviews and meta-analyses on the prevention and intervention of WSL published from January 2011 to June 2021 have been reviewed (Table 4). PubMed was used for this systematic search. The research terms were *enamel demineralization*, *white spot lesion*, *orthodontic, fluoride*, and *prevention*, and their combination. Table 4 provides an overview of the available evidence for the treatment of WSL during multibracket therapy. Topical fluorides are helpful in the prevention of WSL during and after multibracket therapy [33, 67, 68]. Especially the professional application of 12,300 ppm F foam (1.23% acidulated phosphate fluoride (APF)) or varnish in combination with a high fluoride toothpaste (5,000 ppm F) has proven to be the most effective modality (Table 4). This confirms that the sole application of fluoride toothpaste (1,450 ppm F) is not sufficient to prevent enamel demineralization [69, 70]. Nevertheless, the results from Table 4 should be viewed with caution since the available evidence remains limited.

Similar to the Dutch study by Derks et al. [13], a great demand for a guideline was also found in the present study. The most frequently mentioned argument favoring a guideline was the desire for uniformity and systematization. A second argument was that a guideline would serve to make the necessary funding for WSL prevention available. Furthermore, a guideline as an evidence-based tool could also convince and educate reluctant patients and parents. Most practitioners advocate for lingual appliances and preventive measures such as dental prophylaxis over 18 years to be financially covered by the health insurance. Individual risk assessment of enamel demineralization should be considered (type, process, duration, social environment), and personalized measures must therefore be encountered. Consequently, a guideline can merely provide standardized methods to improve oral hygiene during orthodontic treatment, as already tested and implemented in the Netherlands [71, 72].

One shortcoming of this study is the relatively low response rate, which risks that different perspectives did not become apparent. It is quite possible that only those clinicians concerned with the subject voluntarily participated. Nevertheless, participants from 14 out of 16 federal states, including representatives from universities and dental practices with a wide age range, provided information on their strategies for preventing and managing WSL. This diversity strengthens the study’s findings.

The participants of this study seem to have their own approach to preventive strategies. Many participants give instructions on good oral hygiene at the beginning of treatment, but consistent with other studies, these measurements are not carried out regularly [73]. No clear guidance for the treatment of WSL could be encountered in the literature. There is a lack of significant clinical studies, longer follow-ups, and comparisons of intervention methods and daily oral hygiene procedures.

Considering the survey findings and based on the available evidence for clinical practice, we recommend caries risk evaluation [74], repeating oral hygiene instructions combined with virtual interventions/reminders (i.e., mobile phone applications) [38]. Tooth brushing twice daily with fluoridated toothpaste (1,500 ppm) should be instructed, and complemented by an individualized concept for professional tooth cleaning depending on the patient’s oral hygiene, combined with dietary advice. Fluoride varnish should be reapplied at least two times a year or every 4–6 weeks during multibracket therapy in caries susceptible patients [3, 16, 74, 75]. Mouth rinsing twice a day may also be recommended for patients with increased plaque formation [76]. Regarding surface sealant, there is very low evidence of preventing WSL during multibracket therapy [33]. However, the new approach with SAP P11-4 could be promising for preventing WSL [53, 77], applied at the beginning of multibracket therapy and combined with repeated fluoride application [47, 48, 53]. Similar to the bonus program of some health insurance providers in Germany, a points system could also be introduced in orthodontics, and the most diligent patients could be rewarded at the end.

While orthodontists must remain vigilant, a policy framing interaction for primary oral health care would be supportive since caries is still a global health challenge [18, 78]. Especially in the COVID-19 pandemic, access to treatment is impeded, increasing the prevalence of untreated caries [78].

Policy changes from the health insurances are necessary to promote standardized methods, such as regular fluoride varnish application, prophylaxis during multibracket therapy, and in some cases, access to clear aligner or lingual orthodontic appliance [47, 67] (i.e., Molar Incisor Hypomineralization, physical or mental disability, high caries risk). For this reason, a guideline serves to understand better, motivate, and prevent the development of WSL during orthodontic treatment.

The role of oral health care providers is to achieve an overall improvement in oral care. Therefore, all efforts should be made to avoid side effects, such as caries, during orthodontic treatment. Furthermore, prevention programs should be implemented before intervention.

## Conclusion

WSL prevention during multibracket therapy is challenging for orthodontists. Males in puberty are predominantly affected. The results show that the available scientific evidence is not integrated into the routine management of WSL. Younger orthodontists incorporate more than their senior peers’ prevention strategies for WSL during multibracket appliance treatment. Prevention before the intervention, dental health care experience reports, and a practice protocol are recommended.
